# Comparison of High Frequency Positive Pressure Mechanical Ventilation (HFPPV) With Conventional Method in the Treatment of Neonatal Respiratory Failure

**DOI:** 10.5812/ircmj.2791

**Published:** 2013-03-05

**Authors:** Elahe Amini, Fatemeh Sadat Nayeri, Arezu Hemati, Tahere Esmaeilinia, Firuzeh Nili, Hossein Dalili, Majid Aminnejad

**Affiliations:** 1 Materno-Feral and Neonatal Research Center, Valiasr Hospital, Tehran University of Medical Science, Tehran, IR Iran; 2 NICU, Valiasr Hospital, Tehran University of Medical Science, Tehran, IR Iran; 3 Breast Feeding Research Center, Valiasr Hospital, Tehran University of Medical, Tehran, IR Iran

**Keywords:** Respiratory Insufficiency, Infant, Newborn, Ventilation

## Abstract

**Background:**

Respiratory failure is a major problem in neonatal medicine in all over the world and has different causes. Using mechanical ventilation is one of its major treatments.

**Objectives:**

Different strategies have been expressed in this context, including high frequency mechanical ventilation.

**Patients and Methods:**

This study is a prospective randomized clinical trial conducted on all newborns with respiratory failure hospitalized in the NICU of Tehran vali-asr Hospital during 2009.These patients were divided in to two groups through block Randomization method; conventional mechanical ventilation group and high frequency ventilation group.

**Results:**

Intraventricular hemorrhage (IVH) and air leak (e.g. pneumothorax) were less in HFPPV group than conventional group (P = 0.012 and P = 0.038). The mean time needed for mechanical ventilation was lower in HFPPV group, but this difference was not statistically significant (P = 0.922). Needing to O2 in 28 days of age was almost equal in both groups (P = 0. 99). Mortality, and refractory hypoxia and PVL were lower in HFPPV group, but the difference was not statistically significant (P = 0.301, P = 0. 508, P = 0. 113).

**Conclusions:**

Treatment of neonatal respiratory failure with high rate mechanical ventilation may reduce some complications.

## 1. Background

Respiratory failure is the most common cause of neonatal hospitalization in NICU and is expressed as the existence of two or more than two symptoms of the following clinical and laboratory criteria(1). A) Clinical criteria: Retraction (Suprasternal, supraclavicular, intercostals), Grunting, respiratory rate more than 60/min, central cyanosis, refractory apnea, reduced infant activities. B) Laboratory criteria: pacO2 > 60 mmHg , paO2 < 50 mmHg or sat O2 > 80% despite receiving 100% O2, pH > 7.25. Respiratory failure in infants has different intra and extra pulmonary causes (2), and rarely recovers without a ventilation support (1). Several solutions have been proposed for mechanical ventilation in neonates which include conventional mechanical ventilation and HFV that each has advantages and disadvantages. In conventional method, the maximum rate used is 60/min but in HFV method, rate higher than 60/ min is used. In HFV method, very low volume, often less than the anatomic dead space is used with very high rate, and this causes the establishment of adequate gas exchange with low pressure in the lower airways and alveoli (3). In various studies, the effects of this method of mechanical ventilation have been assessed in the treatment of neonatal lung diseases and various results have been obtained (1, 3-7).

## 2. Objectives

Our goal in this study is to compare conventional mechanical ventilation with HFPPV in the desired population and determine its impact on our variables.

## 3. Materials and Methods

This study was a prospective single blinded randomized clinical trial conducted on two groups of 31 patients, during 2009, in the NICU of Tehran Vali-asr Hospital in collaboration with Materno- Feral& Neonatal Research Center. Our study population was all neonates suffering from respiratory failure who were admitted in this ward. In case of complex congenital heart disease, genetic syndromes, and major and contrary to the life anomalies and moderate to severe hypoxic-ischemic Encephalopathy (HIE) or birth asphyxia (5-minute Apgar score 0-3, and PH < 7 in cord blood) the patients did not enter to the study. For all studied infants, Survanta was injected at the dose of 4 cc/kg (maximum 8 cc) and maximum within two hours after birth and then were divided into two groups through Block Randomization methods and placed under mechanical ventilation. In each group, Bear cub 750 was used as mechanical ventilator. Ventilation strategies in each group were based on lung recruitment (for example lung inflation) and prevention of Atelectasis or excessive lung distension. Our definition of Ideal lung inflation was based on the observable of lung air filling till the eighth posterior rib on the neonate’s chest X-ray. In HFPPV group, respiratory rate was adjusted as rate > 60/ min and up to 150/min (based on patient’s need and achievement an acceptable response) and was set with pulse oximetry and ABG. Mean airway pressure (MAP) was considered 2-3 H2O cm higher than the desired MAP in conventional ventilation method, and then we increased MAP 1-2 cm H2O periodically until the achievement of acceptable O2 sat and visible vibration in the infant’s chest and abdomen. Expiratory to inspiratory time ratio was set as 1/3. After reaching the desired oxygenation, we decreased MAP gradually and also reduced Fio_2 _gradually to 40%. Respiratory rate was gradually reduced at the time of reaching MAP to 8-10 H2O cm, actually the patient returned to conventional mechanical ventilation. In case of Fio2 < 40%, PIP = 10-12 cm H2O and Rate = 10-12/min, the patient was ready for extubation. In conventional mechanical ventilation, respiratory rate is maximum 60/min and TV (Tidal Volume) = 4-7 cc / kg and inspiration time is set to 0.25-0.40. The aim was to achieve an acceptable lung distension and appropriate oxygenation in patients. Considering the type of studied patients, acceptable ABG is considered based on the existing reference for each group.

1. RDS→ Po2 = 50-70 mmHg, PH = 7.25-7.35, Pco2 = 45-55mmHg

2. PPHN→ Po2 = 70-100mmHg, PH = 7.35-7.45, Pco2 = 30-40mmHg

### 3.1. Pneumonia and Meconium Aspiration

PH = 7.30-7.40, Po2 = 60-80mmHg, Pco2 = 40-50mmHg. Acceptable pulse oximetry was set between 86-95%. Before mechanical ventilation, CXR was prepared for all patients and 2h and 24h after the start of mechanical ventilation, was repeated. And then, based on clinical conditions, CXR was repeated if necessary. All patients, at the time of requirement for mechanical ventilation, had an umbilical artery or vein catheter and were under permanent monitoring of heart – lung and pulse oximetry during all hospitalization time. Skin temperature, blood pressure and urinary output were checked and recorded every hour. Brain ultrasound was performed for all premature infants (GA < 37 w) on days 0-3, 7 and 14 of birth, and if there were problems such as IVH or periventricle density, brain ultrasonography was repeated at intervals depending on the severity of the lesion. The data were entered in SPSS software version 11.5. We compared quantitative variables (gestational age ,weight, mean duration of mechanical ventilation, mean number of receiving surfactant) by Student's T test and qualitative variables (sex, underlying disease, IVH, air leak, mortality rate, needing to O2 in 28 days, PVL, treatment failure and refractory hypoxia) by Fisher's Exact Test and Pearson chi- square. This study was performed with 80% power and 95% significance level. In order to respect medical ethics, patient parents signed ill-informed and voluntary consent form for their child's participation in the project and were also informed about the potential advantages and disadvantages of the treatment.

## 4. Results

Sixty two patients were entered to the study. Thirty seven patients (59.7%) were male and 25 patients (40.3%) were female. Both groups were matched in demographic characteristics and there was no significant differences between them (Table 1).

**Table 1.** Demographic Characteristics

Variable	Demographic Characteristics		P value
	Conventional Group	HFPPV Group	
**Gestational age, y, Mean ± SD**	32.7 ± 4.2	32.4 ± 4.5	0.773
**Weight, kg, Mean ± SD**	1869.32 ± 883.24	1920 ± 938.36	0.826
**Gender, No.**			0.500
Male	18	19	
Female	13	12	
**Underlying disease, No.**			0.607
RDS	21	21	
PPHD	4	4	
MAS	3	3	
pneumonia	3	3	

Prevalence of IVH and air leak in HFPPV group was less than conventional group (P = 0. 026 and P = 0. 038). In examining other outcomes, no significant difference was observed between two groups (Table 2).

**Table 2.** Outcome of Variables

Variable	Out Come of Variables		P value
	Conventional Group	HFPPV Group	
**IVH, %**	25.8	3.2	0.026
**Air leak, %**	25.8	6.5	0.038
**Mortality rate, %**	19.3	9.7	0.301
**Treatment failure and Refractory hypoxia, %**	22.6	12.9	0.508
**The mean duration of mechanical ventilation, h**	75.5	73.9	0.925
**Mean number of receiving surfactant, frequency**	1.29	1.25	0.808
**Needing to O2 in 28 days, %**	12.9	9.7	0.99
**PVL, %**	12.9	3.2	0.113

## 5. Discussion

Mechanical ventilation is one of the main therapies of neonatal respiratory problems and despite the known beneficial effects; it has its own complications. In this study, two methods of conventional mechanical ventilation and HFPPV were compared in the treatment of neonatal respiratory failure. In our study, the prevalence of air leak in HFPPV group was significantly less than the conventional method group; some other studies have obtained this result as well (3, 6). In some studies, there were not significant differences in the rate of air leak between these two mechanical ventilation methods (4, 5, 7, 8). Different results have been reported in various studies. There was no significant difference in IVH in some of the studies (5, 7-9) and a meta-analysis has reported an increase in the incidence of IVH in the high rate mechanical ventilation group. In Courtney et al. study the rate of chronic pulmonary disease in HFV group was significantly less than conventional ventilation group (10). In a meta-analysis, chronic pulmonary disease was also reported less in HFV method, but the difference was not significant (4). Also in the study conducted by Alice H. Johnson et al. significant differences in the incidence of chronic pulmonary diseases have not been reported in the two groups (9). Some studies reported a significant reduction in neonatal mortality with high rate and low volume ventilation (9, 11). And in some other studies this difference in mortality is not considered (4). Although studies in animal samples indicate the advantage of HFV on conventional ventilation (12-14), but results in human studies, remain unclear (15-18). To answer this question, whether the method of HFV is a better ventilation strategy or not, further studies in larger size sample and longer duration are needed to investigate Long term pulmonary and neuro developmental outcome in these patients.
